# Introducing third-generation periodic table descriptors for nano-qRASTR modeling of zebrafish toxicity of metal oxide nanoparticles

**DOI:** 10.3762/bjnano.15.93

**Published:** 2024-09-10

**Authors:** Supratik Kar, Siyun Yang

**Affiliations:** 1 Chemometrics and Molecular Modeling Laboratory, Department of Chemistry and Physics, Kean University, 1000 Morris Avenue, Union, NJ 07083, USA

**Keywords:** metal nanoparticles, metal oxide nanoparticles, nano-qRASTR, periodic table descriptors, QSAR, zebrafish

## Abstract

Metal oxide nanoparticles (MONPs) are widely used in medicine and environmental remediation because of their unique properties. However, their size, surface area, and reactivity can cause toxicity, potentially leading to oxidative stress, inflammation, and cellular or DNA damage. In this study, a nano-quantitative structure–toxicity relationship (nano-QSTR) model was initially developed to assess zebrafish toxicity for 24 MONPs. Previously established 23 first- and second-generation periodic table descriptors, along with five newly proposed third-generation descriptors derived from the periodic table, were employed. Subsequently, to enhance the quality and predictive capability of the nano-QSTR model, a nano-quantitative read across structure–toxicity relationship (nano-qRASTR) model was created. This model integrated read-across descriptors with modeled descriptors from the nano-QSTR approach. The nano-qRASTR model, featuring three attributes, outperformed the previously reported simple QSTR model, despite having one less MONP. This study highlights the effective utilization of the nano-qRASTR algorithm in situations with limited data for modeling, demonstrating superior goodness-of-fit, robustness, and predictability (*R*^2^ = 0.81, *Q*^2^_LOO_ = 0.70, *Q*^2^_F1_/*R*^2^_PRED_ = 0.76) compared to simple QSTR models. Finally, the developed nano-qRASTR model was applied to predict toxicity data for an external dataset comprising 35 MONPs, addressing gaps in zebrafish toxicity assessment.

## Introduction

Nanomaterials, which are defined as materials that fall in the range of 1–100 nanometers two-dimensionally, are commonly used in the fields of biomedicine, catalysis, and electricity because of their stable and unique performance, small size, and large surface area [1]. Nanomaterials encompass a range of substances that can be categorized as carbon-based, metal oxides, semiconductors, polymers, clays, emulsions, or metals [2]. Metal oxide nanoparticles (MONPs) are metallic oxides that exist within the nanoscale range and can be intentionally created or occur naturally [3]. Under the rapid development of nanotechnology, more and more MONPs including zinc, iron, titanium, and copper are being explored in therapeutic applications such as drug delivery, bioimaging, biosensing, bioelectronics, and tissue engineering applications [4–6]. Simultaneously, many of these particles also presented strong antibacterial, antifungal, antidiabetic, antioxidant, anticancer, and photocatalytic activities [7–9]. Besides the medical field, they are also commonly used in commercial products such as fuel cells and plastics, and environmental applications such as analysis, sensing, remediation, and amendments. However, it is concerning that the environment is affected because of the enormous production and inadvertent use of nanomaterials.

Nanoparticles have been identified in wastewater streams, drinking water sources, and tap water in amounts ranging from nanograms to micrograms per liter [10]. Also, it was reported that MONPs have been found in human tissues such as brain, heart, and liver [11] and that occupational exposure to metal oxide nanomaterials increased oxidative stress biomarkers, suggesting potential DNA oxidative damage and lipid peroxidation [12]. Given the limited data available from human studies, researchers have turned to zebrafish and their embryos for toxicological investigations. Zebrafish embryos are commonly used to identify environmental heavy metal pollution [13]. As a multicellular organism, zebrafish can offer more comprehensive insights into nanomaterials’ kinetics, migration, and transformation than in vitro cell culture assays [14]. Meanwhile, it is considered an equivalent model for investigating developmental toxicity and genotoxicity because around 85% of its genes are comparable to those found in humans [15].

The potential harm to human health posed by newly created MONPs, particularly those used in biomedical applications, necessitates the implementation of safety-by-design strategies for these materials. The potential to lower development timeframes, costs associated with experiments, and late-stage attrition, in addition to ethical, societal, and regulatory pressures to minimize animal testing, make it worthwhile to create computational models that can accurately predict the toxic hazard of novel MONPs before experimental testing and, ideally, before synthesis, based on the intrinsic, synthesis-controlled properties of the MONPs [16–18]. Over the years, QSAR/QSPR/QSTR techniques have been employed to establish correlations between various characteristics of nanomaterials and their toxicity [19–23]. Nano-quantitative read-across structure–toxicity relationship (nano-qRASTR) models are an advanced approach that builds upon the principles of nano-quantitative structure–toxicity relationship (nano-QSTR) models. These models integrate read-across techniques with traditional quantitative structure–activity relationship (QSAR) methods to enhance the predictive capabilities, particularly in datasets with limited data points [19].

Using quantum chemical descriptors, researchers have created several models to evaluate the toxicity of MONPs to different species covering multiple endpoints, and their work has produced significant and trustworthy findings [24–27]. However, significant computational resources and time are needed for the usage of quantum descriptors for modeling purposes. Not only that, but the reproducibility of quantum descriptors is also an issue because of the usage of different quantum methods and basis sets [28–29]. In contrast, periodic table descriptors were derived or directly obtained from the periodic table. They were able to produce models that were comparable to, or even better than, those of quantum-based descriptors in many cases [30–32], which in turn helped to reduce the amount of time needed for computation followed by without using any computational resources.

However, the periodic descriptors of the previous first and second generations have their limitation such as being unable to deal with the influential observations that exist in the present dataset. In this study, we have proposed five third-generation periodic table descriptors along with the application on modeling enzyme inhibition of the zebrafish hatching enzyme ZHE1 with the nano-qRASTR approach to improve the model quality, predictability, and reliability significantly.

## Materials and Methods

### Dataset

The percentage decrease in enzymatic activity expressed in the form of enzyme inhibition to zebrafish in % (%EI_zebrafish_) of the zebrafish hatching enzyme (ZHE1) of 24 MONPs is utilized for the modeling study [33]. The experimental data (%EI_zebrafish_) ranged from −1.04 (Co_3_O_4_) to 44.72 (Cr_2_O_3_).

#### Descriptor calculation

Models were developed based on the fundamental properties of these metal oxides that can be obtained from the periodic table. A total of 28 periodic table descriptors were utilized for nano-QSTR followed by nano-qRASTR modeling. The list of all derived descriptors along with their meaning and symbol is given in Table 1. Periodic table descriptors offer the advantage of rapid acquisition without the need for extensive calculations or software utilization, unlike quantum chemical descriptors. In our earlier work, we have proposed seven and sixteen descriptors, which were classified as first- and second-generation periodic table descriptors, respectively [31,34]. In this study, we have proposed five more periodic table descriptors, termed third-generation periodic table descriptors. These are atomic radius, crystal ionic radii, density of the metal, electron affinity, and ionization energy. The atomic radius is a fundamental property that influences many physical and chemical characteristics of an element. In the context of nanoparticles, the size of the metal atoms directly affects the overall size and surface area of the nanoparticles, which are critical factors in their reactivity and interaction with other materials. The ionic radius is essential for understanding the metal’s behavior in different oxidation states. This is particularly relevant in nanoparticle chemistry, where redox reactions are common. The density of a metal is a macroscopic property that influences the mass and volume of nanoparticles. Electron affinity measures the energy change when an electron is added to a neutral atom, reflecting the tendency of the metal to gain electrons. The first ionization energy is the energy required to remove the outermost electron from a neutral atom, which is a critical factor in determining the metal’s reactivity and stability. For the present study, descriptors of all three generations are computed and employed for modeling. All descriptor values can be found in Supporting Information File 1. Also, an example calculation of all descriptors for Al_2_O_3_ is given in Supporting Information File 1.

**Table 1:** List of periodic table descriptors used for model development.

No.	Generation	Mathematical expression	Description

1	first generation	MW	molecular weight of the metal oxide
2		*N*_metal_	number of metal atoms per molecule
3		*N*_oxy_	number of oxygen atoms per molecule
4		χ	metal electronegativity
5		∑χ	total metal electronegativity in the specific metal oxide
6		∑χ/*n*O	total metal electronegativity in the specific metal oxide relative to the number of oxygen atoms
7		χ_ox_	oxidation number of the metal

8	second generation	*Z*_metal_	atomic number of the metal
9		*Z* ^v^_metal_	number of valence electrons of the metal
10		PN_metal_	period number of the metal
11		λ = (*Z*_metal_ − *Z*^v^_metal_)/*Z*^v^_metal_	core environment of the metal, defined by the ratio of the number of core electrons to the number of valence electrons
12		μ = 1/(PN_metal_ − 1)	—
13		*V*_metal_	valence of the metal
14		α_metal_ = λ·μ	—
15		∑α_metal_ = α_metal_·*N*_metal_	—
16		∑α_oxy_ = *N*_oxy_·0.33	—
17		∑α = ∑α_metal_ + ∑α_oxy_	core count, gives a measure of the molecular bulk
18		ε_metal_ = −α_metal_ + (0.3·*Z*^v^_metal_)	electronegativity count of the metal
19		ε_oxy_ = −α_oxy_ + (0.3·*Z*^v^_oxy_)	electronegativity count of oxygen
20		∑ε = ε_metal_·*N*_metal_ + ε_oxy_·*N*_oxy_	total electronegativity count of the metal oxide
21		∑ε/*N*	summation of epsilon relative to the number of atoms in the molecule
22		(∑α)^2^	square of summation of alpha, gives a measure of molecular bulk
23		(∑ε/*N*)^2^	summation of epsilon divided by the number of atoms squared

24	third generation	*a*_0_	atomic radius of the metal (pm)
25		*r*_ion_	crystal ionic radius of the metal (pm)
26		*d*_metal_	density of the metal (g/cm^3^)
27		Ea	electron affinity (eV)
28		*I*_1_	first ionization energy of the metal (eV)

#### Splitting of the dataset

The selection of training and test sets was based on the principal component analysis score with guaranteed uniform distribution, as we previously reported [34]. In this study, we used the same dataset-splitting method. In our previous study, we removed compound CoO because of outlier behavior that significantly impacted our model quality. However, as we have proposed five new third-generation periodic table descriptors for modeling, in the present study we have included CoO to check the modeling, as well as the prediction capability, of the newly introduced descriptors along with the existing ones. The details of training and test sets can be found in Supporting Information File 1.

#### nano-QSTR model development

The best subset selection (BSS) approach was used to identify the optimal combination of descriptors. The BSS tool can be accessed at https://teqip.jdvu.ac.in/QSAR_Tools/. It systematically evaluates all possible subsets of descriptors to determine the best combination based on a specified criterion, providing a comprehensive search for the most predictive model. This method was preferred over stepwise regression analysis through backward elimination because BSS ensures that the chosen subset is truly optimal by considering all possible models, whereas stepwise regression may overlook some combinations because of its iterative nature. Afterward, the selected descriptors were employed to develop the final model using a multiple linear regression (MLR) statistical tool, which can be accessed at https://teqip.jdvu.ac.in/QSAR_Tools/ [35]. Pearson correlation among descriptors was also checked, which aimed to create a more dependable model and reduce the possibility of intercorrelation among the descriptors.

#### Calculation of RASTR descriptors and development of nano-qRASTR model

RASTR is a method that integrates the ideas of read-across and QSTR for q-RASTR analysis (here we are modeling nanomaterials, hence the term nano-qRASTR) [36]. This method calculates similarity and error-based RASTR descriptors for training and test sets. The RASAR-Desc-Calc-v2.0 tool employs three similarity-based techniques to produce 15 descriptors, namely, SD_Activity, SE, CVact, MaxPos, MaxNeg, Abs Diff, Avg. Sim, SD_Similarity, CVsim, gm (Banerjee-Roy coefficient), gmAvg. Sim, gmSD_Similarity, Pos.Avg.Sim, and Neg.Avg.Sim. These descriptors are essential for identifying structural similarities and predicting biological activity. The tool’s algorithm uses the weighted standard deviation of predicted values, the coefficient of variation of computed predictions, the average similarity level of close training compounds for each query molecule, and other advanced metrics to ensure accurate predictions. Further details about the tool and its features can be found at https://sites.google.com/jadavpuruniversity.in/dtc-lab-software/home [37].

After computing the RASTR descriptors for both the training and test sets, these descriptors were merged with existing periodic table descriptors. Feature selection was then performed using the BestSubsetSelection_v2.1 tool, which can be found at https://teqip.jdvu.ac.in/QSAR_Tools/. This tool produces a comprehensive set of model combinations for a user-specified number of descriptors while ensuring that the intercorrelation does not exceed a certain threshold. The MLR-based nano-qRASTR model was evaluated using the MLRPlusValidation 1.3 software package, which can be found at https://teqip.jdvu.ac.in/QSAR_Tools/.

#### Validation, applicability domain, and *Y*-randomization

The nano-QSTR model and the nano-qRASTR model were validated through measurements of the goodness-of-fit and the internal validation tool of leave-one-out cross-validation (*Q*^2^). The goodness-of-fit of the models was measured using the coefficient of determination (*R*^2^), which indicates how well the model’s predictions match the actual data. Internal validation was performed using the leave-one-out cross-validation (LOO-CV) method:




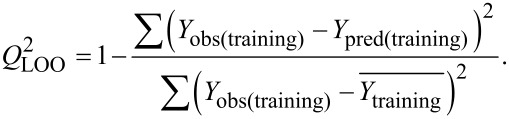




This technique involves removing one data point at a time from the dataset, building the model on the remaining data, and then predicting the excluded data point. The process is repeated for each data point, and the *Q*^2^ metric is calculated to assess the model’s predictive accuracy. Details of the validation metrics can be found in our previous works [17,19,23,36].

We also examined the applicability domain (AD) using the leverage technique to generate the Williams plot [38]. A *Y*-randomization study was also performed to determine if the produced model was generated by chance or not, which entailed performing the model’s calculations 100 times by rearranging the dependent variables while maintaining the original independent variables constant [39]. A *Y*-randomization study has been performed employing “MLR Y-Randomization Test 1.2”, available at https://teqip.jdvu.ac.in/QSAR_Tools/. Following the *Y*-randomization procedure, the study calculated the mean values of *R*^2^ and *Q*^2^ for the 100 randomly generated models.

#### External dataset for data gap filling and prediction reliability

Our prepared external dataset consists of 35 MONPs that were used to predict toxicity for zebrafish. External prediction quality is also checked through the “Prediction Reliability Tool” that employs the AD to our external prediction that is evaluated by three criteria: (1) The mean absolute error is calculated for leave-one-out predictions using the ten most similar training compounds for each query molecule. (2) The standardization approach determines the applicability domain based on similarity. (3) The proximity of the predicted value of the query compound to the experimental mean training response is evaluated [40].

#### Results and Discussion nano-QSTR toxicity model

Equation 1 has been developed employing the BSS-MLR approach for the inhibition of ZHE1 hatching enzyme activity:


[1] $$\begin{array}{c} \%{\text{EI}}_{\text{zebrafish}}=105.05\left(\pm 16.74\right)-5.66\left(\pm 1.94\right)\cdot \sum \chi \\ +\;0.14\left(\pm 0.04\right)\cdot {\left(\sum a\right)}^{2}-0.44\left(\pm 0.08\right)\cdot a_{0}\\ N_{\text{train}}\;=\;16,R^{2}\;=\;0.72,\;R_{\text{adjusted}}^{2}\;=\;0.65,\;Q_{\text{LOO}}^{2}\;=\;0.51;\\ N_{\text{test}}=8,\;R^{2}=0.72,\;Q_{\text{F1}}^{2}=0.72,\;Q_{\text{F2}}^{2}=0.70 \end{array}$$

The first descriptor 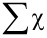 represents the total metal electronegativity in a specific metal oxide and shows a negative correlation to the inhibition of the ZHE 1 hatching enzyme. In this case, an increase in electronegativity will result in a decrease in toxicity. For instance, SnO_2_ has a %EI of 7.12 while having a total metal electronegativity of 3.56. In contrast, the total metal electronegativity of WO_3_ is 1.65, and its observed %EI_zebrafish_ is 42.72. The descriptor 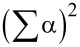 gives a measure of the molecular bulk, which has a positive correlation to the enzyme’s activity. CeO_2_ has an 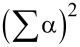 value of 12.50 while it has a %EI value of 2.56; in contrast, TiO_2_ has a 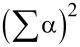 value of 143.76 and a %EI value of 13.28. The last descriptor in our nano-QSTR model is the atomic radius, *a*_o_. The model presents a negative coefficient for the atomic radius (−0.439), suggesting that nanomaterials composed of atoms with larger radii are associated with a decrease in %EI_zebrafish_. A larger atomic radius might indicate weaker bonding and less effective interaction with the enzyme or its substrate, leading to less enzyme inhibition. This could be due to the diffuse nature of the outer electrons in larger atoms, which might reduce the efficiency of electronic interactions essential for binding or catalytic activity.

Our nano-QSTR model suggests that the enzymatic activity of ZHE1 in zebrafish is influenced negatively by the total electronegativity of metals and the atomic radius of the nanomaterial components but positively by the molecular bulk of the nanomaterials. Electronegativity and atomic size determine the reactivity and contact strength of nanomaterials with biological systems, whereas the molecule bulk affects the mechanism of inhibition through steric effects.

#### nano-qRASTR toxicity model

To improve the statistical quality of the nano-QSTR models, we have employed read-across descriptors employing modeled descriptors. Later, all descriptors are merged together and employed for modeling using the BSS-MLR approach. Equation 2 presents the developed nano-qRASTR model:


[2] $$\begin{array}{c} \%{\text{EI}}_{\text{zebrafish}}=-2.01\left(\pm 4.38\right)-0.17\left(\pm 0.06\right)\cdot {\left(\sum \alpha \right)}^{2}\\ +5.10\left(\pm 0.84\right)\cdot \text{SE}\left(\text{LK}\right)\\ -10.93\left(\pm 5.83\right)\cdot \text{CVsim}\left(\text{LK}\right)\\ N_{\text{train}}\;=16,R^{2}\;=0.81,\;R_{\text{adjusted}}^{2}\;=\;0.77,\;Q_{\text{LOO}}^{2}\;=\;0.70;\\ N_{\text{test}}=8,\;R^{2}=0.81,\;Q_{\text{F1}}^{2}=0.76,\;Q_{\text{F2}}^{2}=0.74 \end{array}$$

Like the nano-QSTR model, the nano-qRASTR model also has the 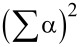 descriptor with a positive contribution to the toxicity. Also, there are two new descriptors from RASTR, namely, SE(LK) and CVsim(LK). “SE” stands for standard uncertainty in the observed response values for the chosen proximate source compounds related to each reference compound. It has a positive contribution to our model with a coefficient of +5.10. The effect of SE(LK) can also be observed in our training set. ZnO has the highest %EI value (42.72) in our training set, while it also has the highest SE(LK) value of 11.47. Conversely, In_2_O_3_ has a SE(LK) value of 2.21, and the experimental %EI value is only 7.12. CVsim(LK), which stands for the coefficient of variation of the similarity values, has a negative contribution to the model. In our dataset, CVsim(LK) did not show a large variation in the values. However, we can observe that Al_2_O_3_ has a relatively large CVsim(LK) value (1.25), while Mn_2_O_3_ has a relatively small CVsim(LK) value of 1.06; their corresponding %EI values are 3.44 and 17.2, respectively.

#### Quality of the nano-qRASTR model

The quality of the nano-qRASTR model was also checked according to the criterion by Golbraikh and Tropsha, with all the metrics falling within the stipulated threshold [41] as follows:




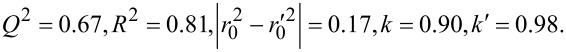




The *Y*-randomization test was also performed to validate if the model was generated by chance. After shuffling all descriptor values, 100 random models were generated. As a result, the average *R*^2^ value is 0.20, while the average *Q*^2^ value is −0.60, which cannot qualify the threshold of 0.5 for both parameters, suggesting that our original model was not developed by chance (details in Supporting Information File 1).

**Figure 1:**
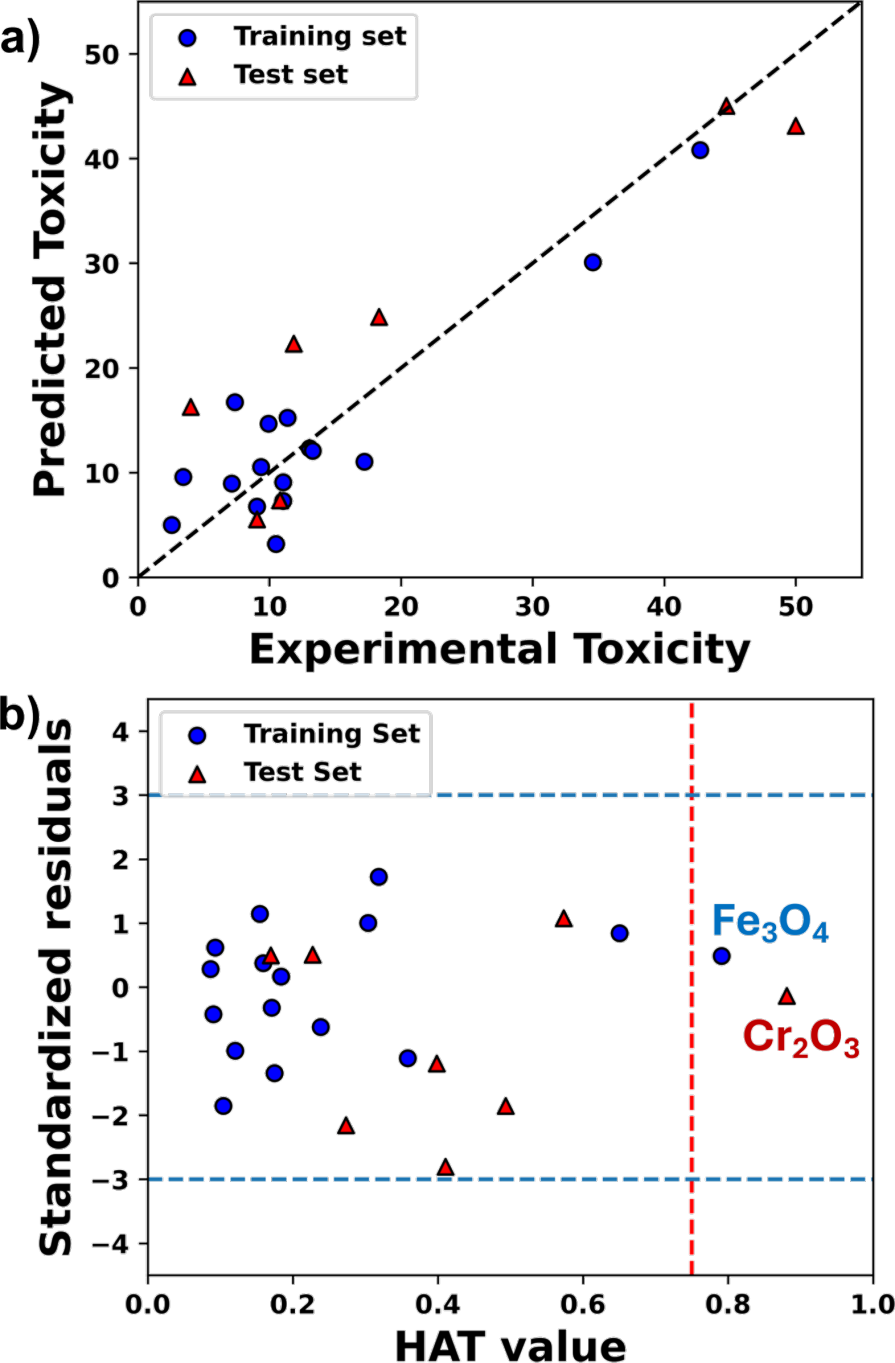
Scatter plot (a) and Williams plot (b) for the nano-qRASTR model. The red dashed line indicates the highest Hat or leverage value, that is, the h* cut-off line.

The scatter plot (Figure 1a) suggests that all MONPs are very close to the best-fit line concerning the experimental toxicity and predicted toxicity values, which further supports the validity of the model. A Williams plot (Figure 1b) was used to verify the prediction reliability by carrying out the applicability domain analysis using the leverage approach. Our result indicates that one training compound (Fe_3_O_4_) is above the leverage critical value. It will be considered as influential *X* outlier. There is also a test date that has a higher value than h* and will be considered as outside of the AD.

**Figure 2:**
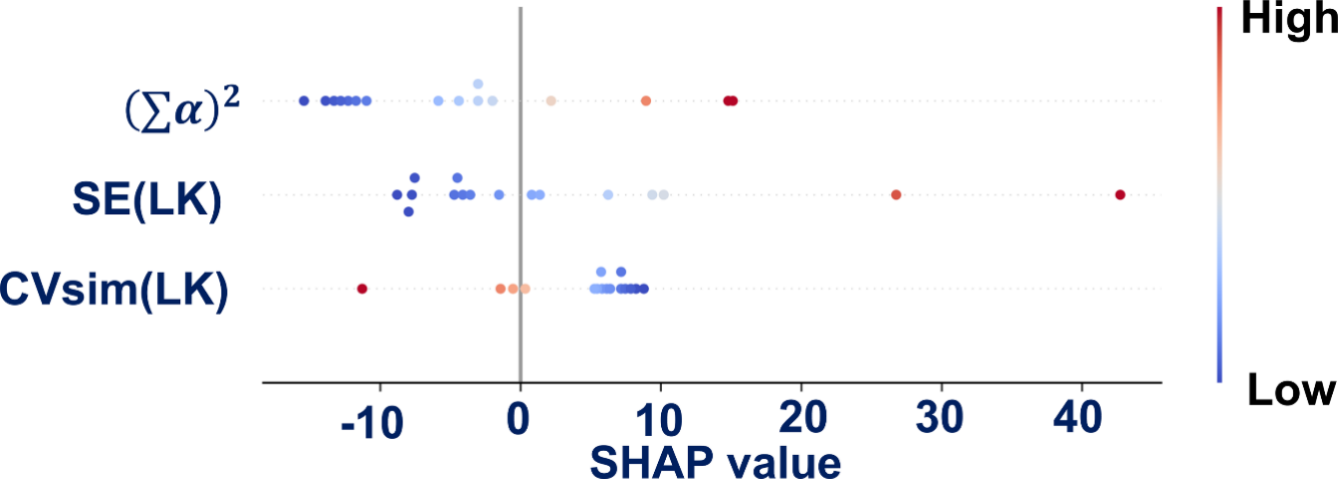
SHAP plot for the nano-qRASTR model.

The SHAP plot (Figure 2) indicates that 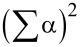 has a predominantly positive effect on the predictions of the model, as the SHAP value increases with increased values of 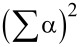. The descriptor SE(LK) shows a more pronounced positive influence on the predicted values. This is consistent with the positive coefficient in our regression equation, and the slight trend from blue to red dots suggests a correlation between feature values and impact. Conversely, CVsim(LK) predominantly affects the model predictions negatively, as evidenced by its SHAP values being mainly on the left side.

#### Mechanisms of ZHE1 enzyme inhibition

**Figure 3:**
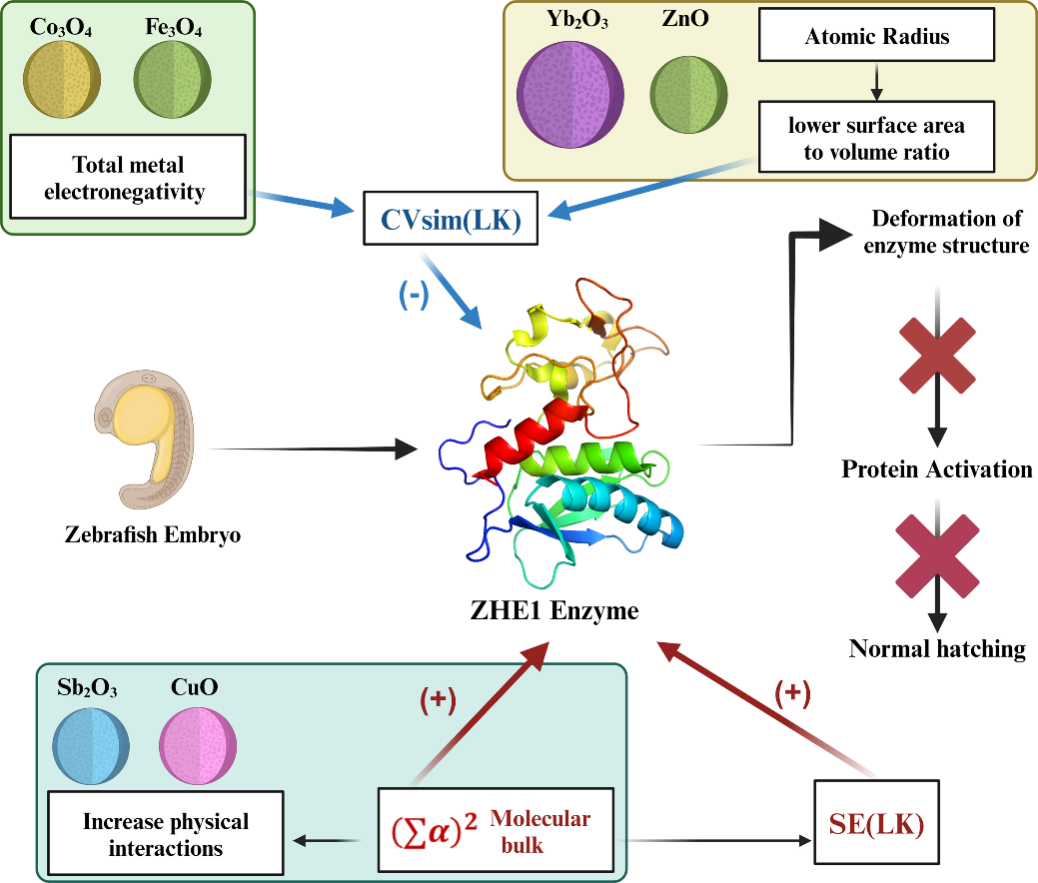
Mechanism of zebrafish hatching enzyme inhibition by MONPs according to the developed models. The figure is “Created with BioRender.com” (https://biorender.com/) with a purchased academic license. This content is not subject to CC BY 4.0.

The incorporation of third-generation descriptors significantly improves the predictive power of the nano-qRASTR model. MONPs with higher metal electronegativity may interfere more strongly with cellular functions of zebrafish, but this does not invariably heighten toxicity; in some instances, it may mitigate oxidative stress and membrane disruption, thereby diminishing toxic effects. Conversely, MONPs with larger atomic radii and crystal ionic radii tend to exhibit a lower surface area-to-volume ratio, which can reduce their cellular interactions and uptake. This reduction in uptake can lead to less cellular dysfunction and toxicity. Larger atomic radii may result in MONPs that are less likely to penetrate cell membranes, thereby decreasing their potential to cause cellular damage and toxicity. However, MONPs with increased molecular bulk can enhance toxicity via several mechanisms. They can physically damage cell membranes, potentially causing cell death. Their size may lead to alternative, more detrimental cellular uptake pathways or provoke harmful responses by accumulating on cell surfaces. Such MONPs might also elevate oxidative stress by triggering the production of reactive oxygen species, which damage cellular components. They can obstruct vital biological processes and, through aggregation, cause localized toxicity to zebrafish. Additionally, their size affects biodistribution and clearance, with larger MONPs tending to accumulate within the zebrafish organism, further exacerbating toxicity (Figure 3). In zebrafish, these mechanisms can manifest in several ways, affecting not only individual cells but also developmental processes. The implications for zebrafish embryos include potential deformities, impaired development, and mortality. Employing zebrafish as a biological model facilitates the evaluation of toxicity, offering an integrative perspective on the hazards that MONPs may present in aquatic ecosystems and living organisms.

#### Comparison with previously published models

Compared to our previous nano-QSTR model (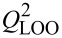 = 0.68, 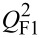 = 0.74, and 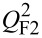 = 0.70) [34], the current nano-qRASTR model demonstrates improvements in these three critical metrics with enhancements of 0.01, 0.02, and 0.05, respectively. Although these improvements might seem minimal, it is crucial to note that in the preceding study, we were able to model 23 MONPs, excluding CoO, which significantly impacted the quality of the model because of its outlier behavior. In contrast, the current study successfully models all 24 MONPs without compromising the model’s quality and predictability, leading to improved results. This suggests that the nano-qRASTR approach is a suitable choice for modeling in cases involving small and complex datasets.

#### External dataset prediction

Predictions for 27 out of 35 MONPs were within the AD, indicating that the nano-qRASTR model confidently predicts 77.14% of the MONPs (Table 2). However, predictions for eight MONPs were considered unreliable as they fell outside the AD. For the MONPs within the AD, the predicted enzyme inhibition (%EI) in zebrafish ranges from 32.42% to 76.16%. Within this spectrum, Ta_2_O_3_ exhibits the highest toxicity, while V_2_O_3_ shows the least.

**Table 2:** Predicted values for an external dataset employing the nano-qRASTR model.

Metal oxide	Modeled descriptors			Predicted %EI_zebrafish_	AD status

	(∑α)^2^	SE(LK)	CVsim (LK)		

Ag_2_O	544.29	8.58	0.73	128.34	out
Au_2_O	994.14	15.14	2.11	225.01	out
Au_2_O_3_	1036.20	15.14	2.11	232.33	out
BaO	32.83	9.87	0.58	47.63	in
BeO	1.77	10.01	0.57	43.14	in
Bi_2_O_3_	52.27	6.58	0.75	32.45	in
CaO	11.09	9.60	0.54	42.89	in
CdO	36.97	9.85	0.43	49.94	in
Co_2_O_3_	86.92	6.49	0.98	35.54	in
Ga_2_O_3_	52.02	6.58	0.73	32.63	in
GeO_2_	8.96	9.97	0.56	44.24	in
HfO_2_	58.68	9.93	0.68	51.40	in
HgO	66.10	8.65	0.33	49.92	in
IrO_2_	84.46	9.00	0.43	53.80	in
MgO	8.01	9.59	0.54	42.28	in
MnO_2_	20.19	9.99	0.54	46.52	iIn
Mo_2_O_3_	461.82	9.80	1.05	116.71	out
Nb_2_O_3_	440.58	9.22	0.87	112.16	out
OsO_2_	64.96	9.45	0.46	52.45	in
PbO	17.89	9.09	0.36	43.42	in
PbO_2_	20.79	9.14	0.35	44.33	in
PdO	0.52	9.58	0.52	41.21	in
PtO	247.43	10.63	1.34	80.54	out
PtO_2_	257.92	10.63	1.34	82.36	out
ReO_2_	63.36	8.74	0.28	50.52	in
Rh_2_O_3_	528.54	11.23	1.36	132.23	out
RuO_2_	130.19	8.92	0.85	56.79	in
Sc_2_O_3_	53.63	7.62	0.34	42.42	in
SrO	23.33	9.88	0.56	46.21	in
Ta_2_O_3_	230.74	9.62	1.00	76.16	in
TcO_2_	33.47	9.66	0.41	48.52	in
Tl_2_O	115.13	7.09	0.57	47.95	in
Tl_2_O_3_	129.73	7.12	0.51	51.25	in
V_2_O_3_	11.49	7.84	0.69	32.42	in
WO_2_	61.78	8.65	0.54	46.92	in

## Conclusion

We have investigated the toxicity of MONPs against zebrafish using a nano-qRASTR model with newly introduced third-generation periodic table descriptors along with first- and second-generation ones. Our results highlight the significance of specific nanoparticle properties influencing the degree of zebrafish toxicity (i.e., the degree of enzyme inhibition), including electronegativity, molecular bulk, and atomic radius of the metal. The developed nano-qRASTR model provides a robust framework for predicting the toxic effects of MONPs based on these fundamental characteristics. Additionally, the introduction of nano-qRASTR model represents a significant methodological enhancement, offering improved predictive accuracy and reliability over previous approaches.

The adoption of third-generation periodic table descriptors has demonstrated that even in the absence of complex quantum chemical calculations, we can achieve high predictive accuracy. This simplification of the descriptor calculation process not only makes the approach more accessible. It also significantly reduces the computational resources required, thus, making it a viable option for rapid screening of nanoparticle toxicity. Our study’s ability to accurately predict the toxicity of a broad range of MONPs to zebrafish highlights its potential as a valuable tool in the safety assessment of nanomaterials. The prediction of 35 diverse MONPs as external dataset also helped to fill the toxicity data gap of zebrafish. The model’s capability to identify compounds with potentially high toxicity offers a pathway to preemptively address the environmental risk assessment and health impacts of nanomaterials. However, only a relatively small number of nanoparticles is included in our training set. While our model shows promising predictive power, the limited diversity and quantity of the training data could restrict the generalizability and robustness of the model. Furthermore, we have only proposed five new third-generation periodic table descriptors. Future work can focus on developing more diverse molecular descriptors with higher effectiveness. Including additional descriptors that capture other critical physicochemical properties could provide a more comprehensive understanding of the mechanisms driving MONP toxicity.

The findings of this study have significant implications for the use of MONPs in medical applications. Nanoparticles are increasingly explored regarding drug delivery, imaging, and therapeutic purposes. Understanding the toxicity mechanisms and predicting potential adverse effects of MONPs can guide the design of safer nanomedicines. MONPs are also being utilized in environmental remediation efforts to remove pollutants from water and soil. The insights gained from this study can help in selecting nanoparticles that are effective in remediation without posing significant risks to aquatic life and ecosystems. For example, nanoparticles with lower toxicity profiles, as predicted by the nano-qRASTR model, can be prioritized for use in environmental cleanup projects. Additionally, the exploration of MONP toxicity through this advanced modeling aligns with the broader goals of sustainable nanotechnology. The nano-qRASTR model aims to reduce the reliance on animal testing by providing a robust in silico method for toxicity prediction, aligning with the ethical goal of reducing animal use in scientific research. By providing a means to predict and mitigate the adverse effects of nanomaterials before they are synthesized and used in applications, this study contributes to the realization of safer nanomaterials production. The complete study is also incorporated into the QSAR model reporting format (QMRF) proposed by the Organization for Economic Cooperation and Development (OECD), which is provided as Supporting Information File 2. The QMRF will offer a standardized framework of the reported q-RASTR models, ensuring consistency and comparability across studies. With detailed documentation of the model, it promotes transparency, helping others understand the model’s assumptions and limitations. The provided QMRF aligns with OECD principles for validation, facilitating regulatory acceptance, and use in decision-making. Additionally, the QMRF will support communication among scientists and regulators, improve model quality by promoting best practices, and aid in the development of non-animal testing methods for chemical safety assessments.

## Supporting Information

File 1 Additional experimental data.

File 2 Content of the study in QMRF format.

## Data Availability

Most of the data are available in the published article and the Supporting Information. Any additional data will be made available from the corresponding author on request.
