# Supplementary material for: Introducing third-generation periodic table descriptors for nano-qRASTR modeling of zebrafish toxicity of metal oxide nanoparticles
Source: Beilstein J Nanotechnol. 2024 Sep 10;15:1142–52. doi: 10.3762/bjnano.15.93 (PMC11406052; doi:10.3762/bjnano.15.93)
Supplement: File 1 — Additional experimental data. [file Beilstein_J_Nanotechnol-15-1142-s001.pdf]

BEILSTEIN JOURNAL OF NANOTECHNOLOGY

Supporting Information

for

Introducing third-generation periodic table descriptors for nano-qRASTR modeling of zebrafish toxicity of metal oxide nanoparticles

Supratik Kar and Siyun Yang

Beilstein J. Nanotechnol. **2024,** *15,* 1142–1152. doi:10.3762/bjnano.15.93

Additional experimental data

License and Terms: This is a supporting information file under the terms of the Creative Commons Attribution License (https://creativecommons.org/licenses/by/4.0). Please note that the reuse, redistribution and reproduction in particular requires that the author(s) and source are credited and that individual graphics may be subject to special legal provisions.
The license is subject to the *Beilstein Journal of Nanotechnology* terms and conditions: (https://www.beilstein-journals.org/bjnano/terms)

Table S1: Detailed description of RASAR descriptors.

RA function	The descriptor *RA function* encapsulates the information from all the different selected structural and physicochemical descriptors.
SD_activity	This is a measure of the dispersion of the response values of the close source congeners for each query compound, measured in terms of weighted standard deviation.
CV_activity	This is the coefficient of variation of the response values of close source compounds
SE	This is the standard error of the response values of the close source congeners for each query compound
Avg.Sim.	This refers to the average similarity values of the close congeners
Pos.Avg.Sim.	This refers to the average similarity values of the close positive/active congeners
Neg.Avg.Sim.	This refers to the average similarity values of the close negative/inactive congeners
SD_similarity	This is the dispersion in the similarity values among the close source congeners
CVsim	This is the coefficient of variation of the similarity values of close source compounds
MaxPos	This represents the similarity of the query compound to the closest positive/active compound
MaxNeg	This represents the similarity of the query compound to the closest negative/inactive compound
AbsDiff	This is an indicator of the absolute difference between the similarities of the query compound to the closest positive source compound and the closest negative source compound.
g_m *[Banerjee-Roy concordance coefficient]*	This identifies the propensity of a query compound to become positive or negative.

Table S2: Detailed molecular descriptors information of the complete dataset (first generation).

Metal oxide	ID	Y	MW	N_{metal}	N_{oxy}	χ	$\sum\chi$	$\sum\chi/nO$	χ_{ox}
AL_2O_3	1	3.44	101.96	2.00	3.00	1.61	3.22	1.07	3.00
CeO_2	2	10.80	172.11	1.00	2.00	1.12	1.12	0.56	4.00
Co_3O_4	3	-1.04	240.80	3.00	4.00	1.88	5.64	1.41	2.67
CoO	4	4.00	74.93	1.00	1.00	1.88	1.88	1.88	2.00
Cr_2O_3	5	44.72	151.99	2.00	3.00	1.66	3.32	1.11	3.00
CuO	6	50.00	79.55	1.00	1.00	1.90	1.90	1.90	2.00
Fe_2O_3	7	11.04	159.69	2.00	3.00	1.83	3.66	1.22	3.00
Fe_3O_4	8	13.04	231.53	3.00	4.00	1.83	5.49	1.37	2.67
Gd_2O3	9	11.36	362.50	2.00	3.00	1.20	2.40	0.80	3.00
HfO_2	10	11.04	210.49	1.00	2.00	1.30	1.30	0.65	4.00
In_2O_3	11	7.12	277.63	2.00	3.00	1.78	3.56	1.19	3.00
La_2O_3	12	13.28	325.81	2.00	3.00	1.10	2.20	0.73	3.00
Mn_2O_3	13	17.20	157.87	2.00	3.00	1.55	3.10	1.03	3.00
NiO	14	34.56	74.69	1.00	1.00	1.91	1.91	1.91	2.00
Ni_2O_3	15	18.32	165.38	2.00	3.00	1.91	3.82	1.27	3.00
Sb_2O_3	16	9.04	291.52	2.00	3.00	2.05	4.10	1.37	3.00
SiO_2	17	10.48	60.08	1.00	2.00	1.90	1.90	0.95	4.00
SnO_2	18	2.56	150.71	1.00	2.00	1.96	1.96	0.98	4.00
TiO_2	19	9.92	79.87	1.00	2.00	1.54	1.54	0.77	4.00
WO_3	20	9.04	231.84	1.00	3.00	2.36	2.36	0.79	6.00
Y_2O_3	21	9.36	225.81	2.00	3.00	1.22	2.44	0.81	3.00
Yb_2O_3	22	11.84	394.08	2.00	3.00	1.10	2.20	0.73	3.00
ZnO	23	42.72	81.38	1.00	1.00	1.65	1.65	1.65	2.00
ZrO_2	24	7.36	123.22	1.00	2.00	1.33	1.33	0.67	4.00

Table S2 (continued): Detailed molecular descriptors information of the complete dataset.

Metal oxide	Z_{metal}	Z^{v}_{metal}	PN_{metal}	λ	μ	V_{metal}	α_{metal}	$\sum\alpha_{metal}$	$\sum\alpha_{oxy}$	$\sum\alpha$	ε_{metal}	ε_{oxy}	$\sum\varepsilon$	$\sum\varepsilon/N$	$(\sum\alpha)^2$	$(\sum\varepsilon/N)^2$
AL_2O_3	13.00	3.00	3.00	3.33	0.50	3.00	1.67	3.33	0.99	4.32	-0.77	1.47	0.70	0.14	18.69	0.02
CeO_2	58.00	2.00	6.00	28.00	0.20	4.00	5.60	5.60	0.66	6.26	-5.00	1.47	-3.53	-1.18	39.19	1.38
Co_3O_4	27.00	2.00	4.00	12.50	0.33	5.00	4.17	12.50	1.32	13.82	-3.57	1.47	-2.10	-0.30	190.99	0.09
CoO	27.00	2.00	4.00	12.50	0.33	5.00	4.17	4.17	0.33	4.50	-3.57	1.47	-2.10	-1.05	20.22	1.10
Cr_2O_3	24.00	1.00	4.00	23.00	0.33	6.00	7.67	15.33	0.99	16.32	-7.37	1.47	-5.90	-1.18	266.45	1.39
CuO	29.00	1.00	4.00	28.00	0.33	4.00	9.33	9.33	0.33	9.66	-9.03	1.47	-7.56	-3.78	93.38	14.30
Fe_2O_3	26.00	2.00	4.00	12.00	0.33	6.00	4.00	8.00	0.99	8.99	-3.40	1.47	-1.93	-0.39	80.82	0.15
Fe_3O_4	26.00	2.00	4.00	12.00	0.33	6.00	4.00	12.00	1.32	13.32	-3.40	1.47	-1.93	-0.28	177.42	0.08
Gd_2O3	64.00	2.00	6.00	31.00	0.20	3.00	6.20	12.40	0.99	13.39	-5.60	1.47	-4.13	-0.83	179.29	0.68
HfO_2	72.00	2.00	6.00	35.00	0.20	4.00	7.00	7.00	0.66	7.66	-6.40	1.47	-4.93	-1.64	58.68	2.70
In_2O_3	49.00	3.00	5.00	15.33	0.25	3.00	3.83	7.67	0.99	8.66	-2.93	1.47	-1.46	-0.29	74.94	0.09
La_2O_3	57.00	2.00	6.00	27.50	0.20	3.00	5.50	11.00	0.99	11.99	-4.90	1.47	-3.43	-0.69	143.76	0.47
Mn_2O_3	25.00	2.00	4.00	11.50	0.33	7.00	3.83	7.67	0.99	8.66	-3.23	1.47	-1.76	-0.35	74.94	0.12
NiO	28.00	2.00	4.00	13.00	0.33	4.00	4.33	4.33	0.33	4.66	-3.73	1.47	-2.26	-1.13	21.75	1.28
Ni_2O_3	28.00	2.00	4.00	13.00	0.33	4.00	4.33	8.67	0.99	9.66	-3.73	1.47	-2.26	-0.45	93.25	0.20
Sb_2O_3	51.00	5.00	5.00	9.20	0.25	5.00	2.30	4.60	0.99	5.59	-0.80	1.47	0.67	0.13	31.25	0.02
SiO_2	14.00	4.00	3.00	2.50	0.50	4.00	1.25	1.25	0.66	1.91	-0.05	1.47	1.42	0.47	3.65	0.22
SnO_2	50.00	4.00	5.00	11.50	0.25	4.00	2.88	2.88	0.66	3.54	-1.68	1.47	-0.21	-0.07	12.50	0.00
TiO_2	22.00	2.00	4.00	10.00	0.33	4.00	3.33	3.33	0.66	3.99	-2.73	1.47	-1.26	-0.42	15.95	0.18
WO_3	74.00	2.00	6.00	36.00	0.20	6.00	7.20	7.20	0.99	8.19	-6.60	1.47	-5.13	-1.28	67.08	1.64
Y_2O_3	39.00	2.00	5.00	18.50	0.25	3.00	4.63	9.25	0.99	10.24	-4.03	1.47	-2.56	-0.51	104.86	0.26
Yb_2O_3	70.00	2.00	6.00	34.00	0.20	3.00	6.80	13.60	0.99	14.59	-6.20	1.47	-4.73	-0.95	212.87	0.89
ZnO	30.00	2.00	4.00	14.00	0.33	2.00	4.67	4.67	0.33	5.00	-4.07	1.47	-2.60	-1.30	24.97	1.69
ZrO_2	40.00	2.00	5.00	19.00	0.25	4.00	4.75	4.75	0.66	5.41	-4.15	1.47	-2.68	-0.89	29.27	0.80

Table S2 (continued): Detailed molecular descriptors information of dataset (third generation).

Metal oxide	a_0	I_1	Ea	d_{metal}	r_{ion}
AL_2O_3	184.00	5.99	0.44	2.70	67.50
CeO_2	235.00	5.54	0.50	6.77	101.00
Co_3O_4	192.00	7.88	0.66	8.86	67.00
CoO	192.00	7.88	0.66	8.86	88.50
Cr_2O_3	189.00	6.77	0.67	7.15	75.50
CuO	140.00	7.73	1.23	8.93	87.00
Fe_2O_3	194.00	7.90	0.16	7.87	69.00
Fe_3O_4	194.00	7.90	0.16	7.87	72.50
Gd_2O3	237.00	6.15	0.21	7.90	107.80
HfO_2	212.00	6.83	0.18	13.30	85.00
In_2O_3	193.00	5.79	0.30	7.31	94.00
La_2O_3	240.00	5.58	0.50	6.15	117.20
Mn_2O_3	197.00	7.43	-0.05	7.30	72.00
NiO	163.00	7.64	1.16	8.91	83.00
Ni_2O_3	163.00	7.64	1.16	8.91	70.00
Sb_2O_3	206.00	8.64	1.07	6.69	90.00
SiO_2	210.00	8.15	1.39	2.33	54.00
SnO_2	217.00	7.34	1.20	7.29	83.00
TiO_2	187.00	6.83	0.08	4.50	74.50
WO_3	210.00	7.98	0.82	19.30	74.00
Y_2O_3	219.00	6.22	0.31	4.47	104.00
Yb_2O_3	242.00	6.25	-0.02	6.90	100.80
ZnO	139.00	9.39	-0.60	7.13	88.00
ZrO_2	186.00	6.63	0.43	6.52	86.00

Table S3: nano-QSTR model and applicability domain information.

ID	Metal oxide	$\sum\chi$	$(\sum\alpha)^2$	a_0	%EI_Zebrafish	Pred %EI_Zebrafish	Outlier Info.
Training set							
1	AL_2O_3	3.22	18.69	184	3.44	8.76	-
7	Fe_2O_3	3.66	80.82	194	11.04	10.74	-
8	Fe_3O_4	5.49	177.42	194	13.04	14.16	-
9	Gd_2O3	2.4	179.29	237	11.36	13.07	-
10	HfO_2	1.3	58.68	212	11.04	13.06	-
11	In_2O_3	3.56	74.94	193	7.12	10.91	-
12	La_2O_3	2.2	143.76	240	13.28	7.81	-
13	Mn_2O_3	3.1	74.94	197	17.2	11.76	-
14	NiO	1.91	21.75	163	34.56	25.83	-
17	SiO_2	1.9	3.65	210	10.48	2.68	-
18	SnO_2	1.96	12.50	217	2.56	0.53	-
19	TiO_2	1.54	15.95	187	9.92	16.57	-
20	WO_3	2.36	67.08	210	9.04	9.13	-
21	Y_2O_3	2.44	104.86	219	9.36	10.12	-
23	ZnO	1.65	24.97	139	42.72	38.29	-
24	ZrO_2	1.33	29.27	186	7.36	20.10	-
Test set							
2	CeO_2	1.12	39.19	235	10.8	1.20	-
3	Co_3O_4	5.64	190.99	192	-1.04	16.13	-
4	CoO	1.88	20.22	192	4	13.06	-
5	Cr_2O_3	3.32	266.45	189	44.72	41.35	Outside AD
6	CuO	1.9	93.38	140	50	46.20	-
15	Ni_2O_3	3.82	93.25	163	18.32	25.21	-
16	Sb_2O_3	4.1	31.25	206	9.04	-4.09	-
22	Yb_2O_3	2.2	212.87	242	11.84	16.80	-

Table S4: nano-qRASTR model and applicability domain information.

ID	Metal oxide	$\sum\chi$	SE(LK)	CVsim(LK)	%EI_Zebrafish	Pred %EI_Zebrafish	Outlier Info.
**Training set**							
1	AL_2O_3	3.22	4.313	1.247	3.44	9.59	-
7	Fe_2O_3	3.66	2.161	1.279	11.04	9.08	-
8	Fe_3O_4	5.49	2.787	2.810	13.04	12.34	Outlier
9	Gd_2O3	2.4	1.364	1.909	11.36	15.26	-
10	HfO_2	1.3	2.385	1.194	11.04	7.30	-
11	In_2O_3	3.56	2.205	1.216	7.12	8.97	-
12	La_2O_3	2.2	1.609	1.748	13.28	12.09	-
13	Mn_2O_3	3.1	2.280	1.059	17.2	11.06	-
14	NiO	1.91	8.339	1.296	34.56	30.10	-
17	SiO_2	1.9	3.245	1.095	10.48	3.19	-
18	SnO_2	1.96	3.358	1.123	2.56	5.00	-
19	TiO_2	1.54	4.930	1.025	9.92	14.68	-
20	WO_3	2.36	1.524	0.975	9.04	6.77	-
21	Y_2O_3	2.44	1.570	1.253	9.36	10.53	-
23	ZnO	1.65	11.470	1.827	42.72	40.81	-
24	ZrO_2	1.33	5.090	1.122	7.36	16.75	-
**Test set**							
2	CeO_2	1.12	2.127	0.757	10.8	7.37	-
3	Co_3O_4	5.64	2.839	2.799	-1.04	15.08	-
4	CoO	1.88	4.149	0.582	4	16.29	-
5	Cr_2O_3	3.32	1.470	0.621	44.72	45.03	Outside AD
6	CuO	1.9	8.398	1.270	50	43.14	-
15	Ni_2O_3	3.82	4.251	1.003	18.32	24.90	-
16	Sb_2O_3	4.1	1.667	0.583	9.04	5.55	-
22	Yb_2O_3	2.2	1.316	1.773	11.84	22.33	-

Table S5: *Y*-randomization results of nano-qRASTR.

MODEL TYPE	R	R^2	Q^2_{LOO}	MODEL TYPE	R	R^2	Q^2_{LOO}
**Original**	**0.90**	**0.81**	**0.69**	Random 31	0.19	0.03	-0.50
Random 1	0.51	0.26	-0.67	Random 32	0.29	0.08	-0.99
Random 2	0.75	0.57	-0.28	Random 33	0.34	0.12	-0.43
Random 3	0.39	0.16	-1.21	Random 34	0.54	0.30	-0.24
Random 4	0.29	0.09	-0.40	Random 35	0.25	0.06	-0.97
Random 5	0.55	0.31	-0.98	Random 36	0.41	0.16	-0.60
Random 6	0.42	0.18	-0.34	Random 37	0.40	0.16	-1.66
Random 7	0.48	0.23	-1.46	Random 38	0.67	0.45	-1.33
Random 8	0.27	0.07	-0.50	Random 39	0.16	0.03	-0.69
Random 9	0.28	0.08	-0.79	Random 40	0.28	0.08	-0.46
Random 10	0.53	0.28	-0.31	Random 41	0.90	0.82	0.52
Random 11	0.18	0.03	-0.43	Random 42	0.58	0.34	-0.23
Random 12	0.26	0.07	-0.50	Random 43	0.65	0.42	-0.68
Random 13	0.43	0.18	-0.83	Random 44	0.26	0.07	-1.33
Random 14	0.38	0.14	-1.18	Random 45	0.34	0.11	-0.41
Random 15	0.28	0.08	-0.34	Random 46	0.40	0.16	-1.88
Random 16	0.65	0.43	0.04	Random 47	0.44	0.19	-1.48
Random 17	0.53	0.28	-0.83	Random 48	0.72	0.52	-0.29
Random 18	0.53	0.28	-0.14	Random 49	0.19	0.04	-0.27
Random 19	0.28	0.08	-0.37	Random 50	0.27	0.07	-0.34
Random 20	0.39	0.15	-0.28	Random 51	0.42	0.18	-0.47
Random 21	0.56	0.32	-0.33	Random 52	0.47	0.22	-1.18
Random 22	0.56	0.31	-0.46	Random 53	0.67	0.45	-0.15
Random 23	0.40	0.16	-0.35	Random 54	0.36	0.13	-0.47
Random 24	0.45	0.20	-0.22	Random 55	0.23	0.05	-0.74
Random 25	0.29	0.08	-0.29	Random 56	0.38	0.15	-0.61
Random 26	0.23	0.05	-0.71	Random 57	0.47	0.22	-0.80
Random 27	0.28	0.08	-0.87	Random 58	0.33	0.11	-0.64
Random 28	0.58	0.33	-0.95	Random 59	0.38	0.15	-0.46
Random 29	0.55	0.30	-0.18	Random 60	0.17	0.03	-0.50
Random 30	0.36	0.13	-0.42	Random 61	0.61	0.37	-0.22

Random 62	0.41	0.17	-0.23	Random 82	0.64	0.41	-0.28
Random 63	0.59	0.35	-1.21	Random 83	0.50	0.25	-0.50
Random 64	0.30	0.09	-1.14	Random 84	0.30	0.09	-0.27
Random 65	0.26	0.07	-0.37	Random 85	0.37	0.14	-1.23
Random 66	0.23	0.05	-0.49	Random 86	0.26	0.07	-0.38
Random 67	0.32	0.10	-0.41	Random 87	0.60	0.36	-0.01
Random 68	0.42	0.18	-0.64	Random 88	0.22	0.05	-0.67
Random 69	0.55	0.30	-0.43	Random 89	0.48	0.23	-0.16
Random 70	0.58	0.34	-0.62	Random 90	0.34	0.12	-1.05
Random 71	0.73	0.53	-0.06	Random 91	0.22	0.05	-0.47
Random 72	0.46	0.21	-1.32	Random 92	0.25	0.06	-1.00
Random 73	0.48	0.23	-0.41	Random 93	0.50	0.25	-1.18
Random 74	0.34	0.12	-1.19	Random 94	0.37	0.14	-1.23
Random 75	0.56	0.32	-0.48	Random 95	0.16	0.03	-0.44
Random 76	0.57	0.32	-1.67	Random 96	0.29	0.09	-0.27
Random 77	0.76	0.58	0.17	Random 97	0.25	0.06	-1.24
Random 78	0.33	0.11	-0.63	Random 98	0.42	0.18	-1.11
Random 79	0.37	0.14	-0.28	Random 99	0.26	0.07	-0.49
Random 80	0.46	0.21	-0.37	Random 100	0.21	0.04	-0.42
Random 81	0.40	0.16	-0.50				

Table S6: Example calculation for periodic table descriptors with Al_2O_3 as example.

Descriptor	Calculation	Value
MW	Obtained from the Periodic Table	101.96
N_{metal}	Obtained directly from chemical formula	2
N_{oxy}	Obtained directly from chemical formula	3
χ	Obtained directly from the Periodic Table	1.61
$\sum\chi$	$N_{metal} \times \chi = 1.61 \times 2$	3.22
$\sum\chi$/nO	$\frac{\sum\chi}{N_{oxy}} = \frac{3.22}{3}$	1.073
χ_{ox}	$\frac{N_{oxy} \times 2}{N_{metal}} = \frac{3 \times 2}{2}$	3
Z_{metal}	Obtained directly from the Periodic Table	13
Z^v_{metal}	Obtained directly from the Periodic Table	3
PN_{metal}	Obtained directly from the Periodic Table	4
$\lambda=(Z_{metal}- Z^v_{metal})/Z^v_{metal}$	$\frac{Z_{metal}- Z^v_{metal}}{Z^v_{metal}} = \frac{13-3}{3}$	3.33

$\mu = 1/(PN_{metal}-1)$	$\frac{1}{PN_{metal}-1} = \frac{1}{3-1}$	0.5
V_{metal}	Obtained directly from the Periodic Table	3
$\alpha_{metal} = \lambda * \mu$	$\lambda \times \mu = 3.33 \times 0.5$	1.67
$\sum\alpha_{metal} = \alpha_{metal} * N_{metal}$	$\alpha_{metal} \times N_{metal} = 1.67 * 2$	3.33
$\sum\alpha_{oxy} = N_{oxy}*0.33$	$N_{oxy} \times 0.33 = 3 * 0.33$	0.99
$\sum\alpha = \sum\alpha_{metal} + \sum\alpha_{oxy}$	$\sum\alpha_{metal} + \sum\alpha_{oxy} = 3.33 + 0.99$	4.32
$\varepsilon_{metal} = -\sum\alpha_{metal} + (0.3*Z^{v}_{metal})$	$-\alpha_{metal} + (0.3 \times Z^{v}_{metal}) = -1.67 + (0.3 \times 3)$	-0.77
$\varepsilon_{oxy} = -\alpha_{oxy} + (0.3*Z^{v}_{oxy})$	$-\alpha_{oxy} + \left(0.3 * Z^{v}_{oxy}\right) = -0.99 + (0.3 \times 6)$	0.81
$\sum\varepsilon = \varepsilon_{metal} * N_{metal} + \varepsilon_{oxy} * N_{oxy}$	$\varepsilon_{metal} + \varepsilon_{oxy} = -2.43 + 0.81$	-1.62
$\sum\varepsilon/N$	$\frac{\sum\varepsilon}{N} = -\frac{1.62}{2+3}$	-0.32
$(\sum\alpha)^2$	$(\sum\alpha)^2 = 4.32^2$	18.69
$(\sum\varepsilon/N)^2$	$\left(\frac{\sum\varepsilon}{N}\right)^2 = -0.32^2$	0.105
a_0	Obtained directly from the Periodic Table	184.00
r_{ion}	Obtained directly from the Periodic Table	5.99
d_{metal}	Obtained directly from the Periodic Table	0.44
Ea	Obtained directly from the Periodic Table	2.70
I_1	Obtained directly from the Periodic Table	67.50
