# Supplementary material for: Introducing third-generation periodic table descriptors for nano-qRASTR modeling of zebrafish toxicity of metal oxide nanoparticles
Source: Beilstein J Nanotechnol. 2024 Sep 10;15:1142–52. doi: 10.3762/bjnano.15.93 (PMC11406052; doi:10.3762/bjnano.15.93)
Supplement: File 2 — Content of the study in QMRF format. [file Beilstein_J_Nanotechnol-15-1142-s002.pdf]

BEILSTEIN JOURNAL OF NANOTECHNOLOGY

Supporting Information

for

Introducing third-generation periodic table descriptors for nano-qRASTR modeling of zebrafish toxicity of metal oxide nanoparticles

Supratik Kar and Siyun Yang

Beilstein J. Nanotechnol. **2024,** *15,* 1142–1152. doi:10.3762/bjnano.15.93

Content of the study in QMRF format

License and Terms: This is a supporting information file under the terms of the Creative Commons Attribution License (https://creativecommons.org/licenses/by/4.0). Please note that the reuse, redistribution and reproduction in particular requires that the author(s) and source are credited and that individual graphics may be subject to special legal provisions.
The license is subject to the *Beilstein Journal of Nanotechnology* terms and conditions: (https://www.beilstein-journals.org/bjnano/terms)

Quantitative Structure–Activity Relationship (QSAR) Model Reporting Format (QMRF)

1. QSAR Model Identification

1.1. QSAR identifier (title)
Enhancing Nano-qRASTR Modeling of Zebrafish Toxicity of Metal Oxide Nanoparticles using Third-Generation Periodic Table Descriptors

1.2 Other related models
None

1.3. Software coding the model
BSS-MLR v2.1, MLRPlusValidation 1.3, RASAR-Desc-Calc-v2.0

2. General information

2.0 Abstract
This study develops and enhances predictive models for zebrafish toxicity assessment of metal oxide nanoparticles (MONPs). By integrating traditional QSAR techniques with read-across approaches, the nano-qRASTR model addresses data gaps and improves the reliability of toxicity predictions.

2.1. Date of QMRF
26 July 2024

2.2. QMRF author(s) and contact details
Dr. Supratik Kar, Department of Chemistry, Kean University, skar@kean.edu

2.3. Date of QMRF update(s)
N/A

2.4. QMRF update(s)
N/A

2.5. Model developer(s) and contact details
Dr. Supratik Kar, Department of Chemistry, Kean University, skar@kean.edu

2.6. Date of model development and/or publication
2024

2.7. Reference(s) to main scientific papers and/or software package
- Kar, S.; Gajewicz, A.; Puzyn, T.; Roy, K.; Leszczynski, J. Ecotoxicol. Environ. Saf. 2014, 107, 162-169.
- De, P.; Kar, S.; Roy, K.; Leszczynski, J. Environ. Sci. Nano 2018, 5, 2742-2760.

- Khan, K.; Khan, P. M.; Lavado, G.; Valsecchi, C.; Pasqualini, J.; Baderna, D.; Marzo, M.; Lombardo, A.; Roy, K.; Benfenati, E. Chemosphere 2019, 229, 8-17.
- Yang, S.; Kar, S. Science of The Total Environment 2024, 907, 167991.
- Banerjee, A.; Roy, K. Mol. Divers. 2022, 26, 2847-2862.

2.8. Availability of information about the model
The model is non-proprietary. Full description of the model algorithm, training, and test sets are available as supplementary material of the original research article.

2.9. Availability of another QMRF for exactly the same model
None

3. Defining the endpoint - OECD Principle 1: "A DEFINED ENDPOINT"

3.1. Species
Zebrafish (Danio rerio)

3.2. Endpoint
Enzyme inhibition (%EI_Zebrafish) of zebrafish hatching enzyme (ZHE1)

3.3 Comment on endpoint
Zebrafish embryos are used to evaluate the toxicity of MONPs. The endpoint measures the percentage decrease in enzymatic activity of ZHE1.

3.4. Endpoint units
Percentage (%)

3.5. Dependent variable
%EI_Zebrafish

3.6. Experimental protocol
The experimental protocol followed OECD guidelines for toxicological studies in zebrafish embryos. All data are collected from literature as mentioned in the article.

3.7. Endpoint data quality and variability
Data quality was ensured through repeatability and reproducibility checks. The purity of test chemicals was maintained at high levels to ensure accurate correlations between structures and effects. All data are collected from literature as mentioned in the article.

4. Defining the algorithm - OECD Principle 2: "AN UNAMBIGUOUS ALGORITHM"

4.1. Type of model
Equation-based, multiple linear regression (MLR)

4.2. Explicit algorithm
qRASTR model:

$$\%EI_{Zebrafish} = -2.01(\pm 4.38) + 0.17(\pm 0.06) * (\sum\alpha)^2 + 5.10(\pm 0.84) * SE(LK) - 10.93(\pm 5.83) * CVsim(LK)$$

4.3. Descriptors in the model

$\sum\alpha^2$ (Summation of squared alpha), SE(LK) (Standard error of the response values), CVsim(LK) (Coefficient of variation of the similarity values)

4.4. Descriptor selection

Descriptors were selected using the Best Subset Selection (BSS) approach, evaluating all possible subsets of descriptors to determine the best combination.

4.5. Algorithm and descriptor generation

Descriptors were derived from periodic table properties and computed using the RASAR-Desc-Calc-v2.0 tool.

4.6. Software name and version for descriptor generation

RASAR-Desc-Calc-v2.0, BSS-MLR v2.1, MLRPlusValidation 1.3

4.7. Chemicals/Descriptors ratio

16 chemicals (training set) / 3 descriptors = 5.33

5. Defining the applicability domain - OECD Principle 3: "A DEFINED DOMAIN OF APPLICABILITY"

5.1. Description of the applicability domain of the model

The model's applicability domain was defined using the leverage approach, identifying structural features, descriptor space, and response space for reliable predictions.

5.2. Method used to assess the applicability domain

Williams plot was used to assess the applicability domain, with leverage critical value identifying influential observations and outliers.

5.3. Software name and version for applicability domain assessment

MLRPlusValidation 1.3

5.4. Limits of applicability

The model is applicable to MONPs with similar structural and physicochemical properties to those in the training set.

6. Defining goodness-of-fit and robustness (internal validation) – OECD Principle 4: "APPROPRIATE MEASURES OF GOODNESS-OF-FIT, ROBUSTNESS AND PREDICTIVITY"

6.1. Availability of the training set

The training set is available and attached as supporting information.

6.2. Available information for the training set
MONPs name, Descriptor values, Dependent variable values

6.3. Data for each descriptor variable for the training set
Available and attached as supporting information.

6.4. Data for the dependent variable for the training set
Available and attached as supporting information.

6.5. Other information about the training set
16 MONPs were selected based on their relevance and data availability.

6.6. Pre-processing of data before modelling
N/A

6.7. Statistics for goodness-of-fit
R^2 = 0.81, R^2adjusted = 0.77

6.8. Robustness - Statistics obtained by leave-one-out cross-validation
Q^2_LOO = 0.70

6.9. Robustness - Statistics obtained by leave-many-out cross-validation
N/A

6.10. Robustness - Statistics obtained by Y-scrambling
Average R^2 = 0.20 and Average Q^2 = -0.60 of random 100 models

6.11. Robustness - Statistics obtained by bootstrap
N/A

6.12. Robustness - Statistics obtained by other methods
N/A

7. Defining predictivity (external validation) – OECD Principle 4: "APPROPRIATE MEASURES OF GOODNESS-OF-FIT, ROBUSTNESS AND PREDICTIVITY"

7.1. Availability of the external validation set
The external validation set (test set) is available and attached as supporting information.

7.2. Available information for the external validation set
MONPs name, Descriptor values, Dependent variable values

7.3. Data for each descriptor variable for the external validation set
Available and attached as supporting information.

7.4. Data for the dependent variable for the external validation set
Available and attached as supporting information.

7.5. Other information about the external validation set
8 MONPs were included for external validation (test set).

7.6. Experimental design of test set
Randomly selected from available MONPs data.

7.7. Predictivity - Statistics obtained by external validation
$R^2 = 0.81$, $Q^2_{F1} = 0.76$, $Q^2_{F2} = 0.74$

7.8. Predictivity - Assessment of the external validation set
The external validation set (test set) is representative of the training set's descriptor and response space.

7.9. Comments on the external validation of the model
The model demonstrates high predictivity for an external validation (test set) comprising 8 MONPs.

8. Providing a mechanistic interpretation - OECD Principle 5: "A MECHANISTIC INTERPRETATION, IF POSSIBLE"

8.1. Mechanistic basis of the model
The model's mechanistic basis is the interaction of MONPs with biological systems influenced by their electronegativity, molecular bulk, and atomic radius. Incorporating third-generation descriptors enhances the predictive power of the nano-qRASTR model. MONPs with higher metal electronegativity may bind strongly to zebrafish, potentially interfering with cellular functions, but this can also mitigate oxidative stress and membrane disruption, reducing toxicity. Conversely, MONPs with larger atomic and crystal ionic radii have a lower surface area-to-volume ratio, reducing cellular interactions and uptake, thus lessening toxicity. Larger atomic radii may prevent MONPs from penetrating cell membranes, decreasing potential damage. However, increased molecular bulk in MONPs can enhance toxicity by physically damaging cell membranes, triggering reactive oxygen species (ROS) production, and obstructing vital biological processes, leading to localized toxicity and accumulation within zebrafish, worsening toxicity.

8.2. A priori or a posteriori mechanistic interpretation
A posteriori mechanistic interpretation was conducted based on model results and descriptor analysis.

8.3. Other information about the mechanistic interpretation
Higher metal electronegativity and larger atomic radii decrease toxicity, while increased molecular bulk enhances toxicity through mechanisms like ROS production and cellular uptake.

9. Miscellaneous information

9.1. Comments
The model shows significant improvements over previous nano-QSTR models and can be used to predict the toxicity of new MONPs before experimental testing. For the toxicity data gap of MONPs, 35 MONPs were collected as a true external set and predicted toxicity in zebrafish, with

27 predictions within the Applicability Domain (AD), yielding a 77.14% reliability. For these MONPs, enzyme inhibition (%EI) ranged from 32.42% to 76.16%. Ta_2O_3 exhibited the highest toxicity, while V_2O_3 showed the least. Eight MONPs were deemed unreliable, falling outside the AD.

9.2. Bibliography

- Basant, N.; Gupta, S. Nanotoxicology 2017, 11, 339-350.
- Kar, S.; Gajewicz, A.; Roy, K.; Leszczynski, J.; Puzyn, T. Ecotoxicol. Environ. Saf. 2016, 126, 238-244.
- Thwala, M. M.; Afantitis, A.; Papadiamantis, A. G.; Tsoumanis, A.; Melagraki, G.; Dlamini, L. N.; Ouma, C. N. M.; Ramasami, P.; Harris, R.; Puzyn, T.; Sanabria, N.; Lynch, I.; Gulumian, M. *Struct. Chem.* **2022**, *33*, 527-538.
- Lin, S.; Zhao, Y.; Ji, Z.; Ear, J.; Chang, C. H.; Zhang, H.; Low-Kam, C.; Yamada, K.; Meng, H.; Wang, X.; Liu, R.; Pokhrel, S.; Mädler, L.; Damoiseaux, R.; Xia, T.; Godwin, H.A.; Lin, S.; Nel, A.E. *Small* **2013**, *9*, 1776-1785.

9.3. Supporting information

Names of the MONPs, descriptor values, and response values for the training, external validation (test sets) and true external set are provided under supplementary material.
